# Does perioperative electroacupuncture reduce postoperative pain in dogs undergoing ovariohysterectomy?

**DOI:** 10.3389/fvets.2024.1513853

**Published:** 2025-01-07

**Authors:** Celine Iwe, Anika Schiele, Vanessa Passenegg, Daniele Corona, Regula Bettschart-Wolfensberger, Vanessa Heitzmann

**Affiliations:** Department for Clinical Diagnostics and Services, Section of Anaesthesiology, Vetsuisse Faculty, University of Zurich, Zurich, Switzerland

**Keywords:** multimodal analgesia, electroacupuncture, ovariohysterectomy, canine, pain score

## Abstract

**Introduction:**

This study aimed to investigate the analgesic efficacy of perioperative electroacupuncture in fifty-six healthy female dogs undergoing ovariohysterectomy as part of a catch-neuter-release project.

**Materials and methods:**

Ten minutes after sedation with 20 μg/kg medetomidine combined with 0.3 mg/kg butorphanol intramuscularly, the dogs were randomly allocated into two groups and received either electroacupuncture (EA, *n* = 27) or sham acupuncture (C, *n* = 29) treatment for 10 min (after sedation until the end of the surgery) at 6 different acupuncture points LI-4 (Large intestine 4), LIV-3 (Liver 3), ST-36 (Stomach 36), SP-6 (Spleen 6) bilateral. After administration of 0.2 mg/kg meloxicam and anesthesia induction with 2 mg/kg ketamine intravenously, ovariohysterectomy was performed by the same experienced surgeon using a midline surgical approach in dorsal recumbency. Pain was evaluated by two blinded and independent anesthetists using the Short Form of the Multimodal Glasgow Composite Pain Scale (mCMPS-SF) before sedation (T0), and three (T3), six (T6), and 24 h (T24) after anesthesia induction.

**Results:**

Postoperative pain scores did not differ significantly between the groups (*p* = 0.36), but increased significantly three (T3) (*p* = 0.001) and six (T6) (p = 0.001) hours after surgery compared to before sedation and 24 h postoperative in both groups. Three hours after surgery (T3), 89.4% and six hours postoperatively (T6), 71.4% of the dogs in both groups exceeded the analgesic intervention threshold of the mCMPS-SF, indicating the need for rescue analgesia.

**Discussion:**

The results of the study indicate that perioperative electroacupuncture treatment did not improve postoperative pain in dogs undergoing ovariohysterectomy. Therefore, a 10-min electroacupuncture treatment may be insufficient to provide effective postoperative analgesia. The pain assessment in feral dogs was notably impacted by anxious behavior, which may have influenced the final outcome The pain threshold was exceeded in ¾ of the dogs in the early postoperative phase (T3, T6), suggesting that the widely used anesthesia protocol consisting of butorphanol, ketamine and medetomidine in combination with meloxicam may not provide long-lasting and sufficient pain relief.

## 1 Introduction

The worldwide total population of domestic dogs is estimated at approximately 700 million to 1 billion. Around 75% of them are categorized as free-roaming dogs, that are unrestricted in free movement and reproduction. To restrain uncontrolled propagation of animal numbers and prevent exponential growth of populations, several organizations, such as the “World Organization for Animal Health,” developed dog population management programs (1), and determined neutering of female individuals, using ovariohysterectomy and ovariectomy, as the most effective method for reproductive restriction (2, 3).

It is known that these interventions cause acute pain and need to be treated with a multimodal analgesia approach (4, 5). The use of combined analgesics, with different pharmacological mechanisms of action instead of single-drug treatments, was shown to be more effective (6, 7) and has gained broad acceptance in veterinary medicine (8). Further, the occurrence of negative side effects is reduced, as lower dosages are required when analgesic drugs are combined (5, 6).

A commonly used anesthesia protocol, in private practice as well as in neuter programs for elective surgical procedures, is the combination of medetomidine, butorphanol, and ketamine (9, 10). Although the combination of alpha_2_-agonists, opioids and ketamine provides an antinociceptive synergism, besides their sedative effects (11, 12), recently an observational study, which developed a welfare assessment protocol for catch-neuter-release programs (CNR) identified an insufficient postoperative pain management as one of the most common welfare issues occurring in course of CNR-projects (13). To enhance postoperative pain management, incorporating complementary medicine, such as acupuncture, is a valuable option. These treatments are widely accepted and appreciated by owners and have the additional benefit of reducing the adverse effects associated with pharmacological treatments (14, 15).

In veterinary medicine acupuncture has gained growing recognition as part of a complementary treatment to improve pain management, since the AAHA/AAFP (American Animal Hospital Association/American Association of Feline Practitioners) Pain Management Guidelines for Dogs and Cats described acupuncture as an helpful adjunct treatment for postoperative pain following ovariohysterectomy in cats and dogs and for managing intervertebral disc disease (5). Acupuncture provides inflammatory, as well as neuropathic and visceral analgesia via three mechanisms: peripheral, spinal and supraspinal. On the peripheral level, a stimulation of sympathetic nerve fibers promotes the release of *β*-endorphin and encephalin from immune cells (16–18), while the activation of the hypothalamus-pituitary–adrenal axis leads to increased levels of corticosterone and the inhibition of proinflammatory cytokines (19–21). At spinal level opioids, norepinephrine and serotonin are involved in attenuating the nociceptive transmission to supraspinal structures (22, 23), whereas an induced release of endorphins in several nuclei located in the upper brain stem cause a suppression of GABA release and inhibit the affective pain perception in brain areas that are involved in the recognition and memory of painful events, as well as for autonomic and behavioral responses to pain. Further, electrical stimulation at certain acupoints influences the innate and adaptive immune system and supports anti-inflammatory responses by regulating immune cells and cytokines release (24, 25). Compared to dry needle acupuncture, electroacupuncture, defined as an application of electrical stimuli to acupuncture needles, is considered to obtain faster and greater analgesic effects (26), enhances postoperative pain management and decreases anesthetic and opioid requirements (27, 28).

This study aimed to investigate the analgesic efficacy of perioperative electroacupuncture in female dogs undergoing ovariohysterectomy as part of a catch-neuter-release project. The hypothesis of this study was that the use of electroacupuncture in dogs undergoing elective ovariohysterectomy (OVH) decreases postoperative pain scores.

## 2 Materials and methods

### 2.1 Experimental design

This prospective, experimental, randomized, and blinded study was ethically approved by the Cyprus Turkish Veterinarians Medical Association and the local Animal Welfare Officer.

To investigate the postoperative analgesic efficacy of perioperative electroacupuncture in female dogs undergoing ovariohysterectomy, the dogs were randomly allocated to either the electroacupuncture group (EA) or the control group (C), using lots in a blinded envelope as stratified block randomization (blocks of 10 dogs). Following the ovariohysterectomy the postoperative pain was evaluated three (T3), six (T6) and 24 h (T24) after anesthesia induction.

### 2.2 Animals

Seventy-one (EA, *n* = 34), (C, *n* = 37) feral female intact, crossbreed dogs (> 6 months, estimated based on teeth age examination) underwent elective ovariohysterectomy at Girne shelter in North Cyprus as part of a trap-neuter-return mission by the “Network for Animal Protection.” Based on a short physical examination, including heart and lung auscultation, evaluation of mucous membranes, abdominal palpation and rectal body temperature, only dogs which were determined to be clinically healthy, classified as ASA 1 according to the American Society of Anesthesiologists (29), were included in the study. Physiological parameters, such as heart and lung auscultation, mucous membranes, and rectal body temperature of these dogs were all within normal limits. Exclusion criteria were aggressive behavior, which would have required a higher dosage of sedation to be able to perform acupuncture, dogs with a body weight below 5 kg, the presence of any systemic or localized diseases (cardiovascular, respiratory, neurological, or urogenital diseases) and any changes in the uterus (pyometra, mucometra), as well as late pregnancy (> 3^rd^ trimester).

### 2.3 Anesthesia standard protocol

**Figure 1:**
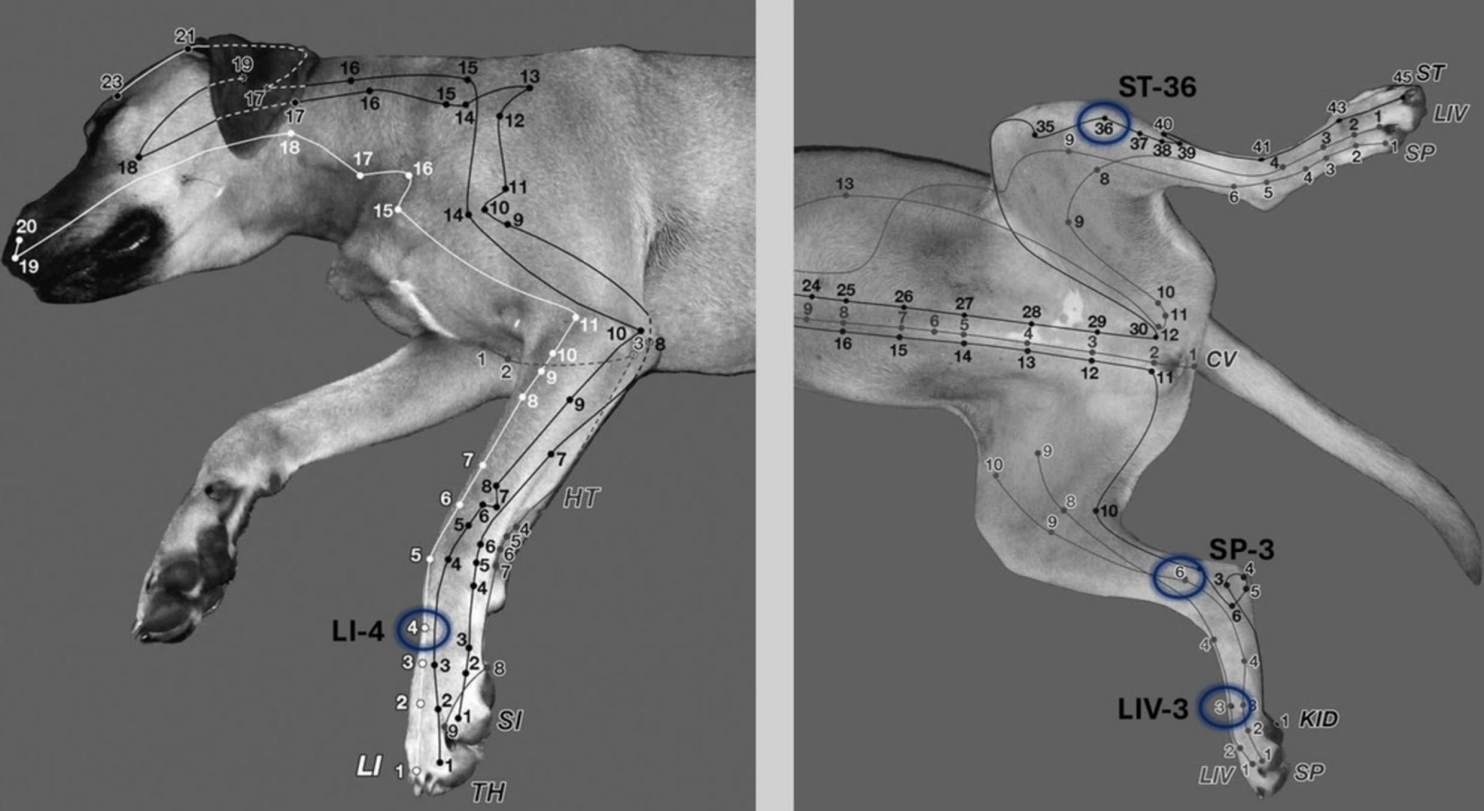
In the electroacupuncture group the following acupoints were stimulated bilaterally to improve postoperative pain in dogs undergoing ovariohysterectomy: LI-4 (Large intestine 4), LIV-3 (Liver 3), ST-36 (Stomach 36), SP-6 (Spleen 6). 1 cun: The width of the last rib or the first tail vertebra is one cun. LI-4: Between the 2nd and 3rd metacarpal bones, at the midpoint of the 3rd metacarpal bone on the medial side. LIV-3: Between the 2nd and 3rd metatarsal bones, proximal to the metatarsophalangeal joint. ST-36: 0.5 cun lateral to the cranial crest of the tibia; in the belly of the cranial tibialis muscle (a long linear point). SP-6: 3 cun proximal to the tip of the medial malleolus in a small depression on the caudal border of the tibia (31).

All dogs were fasted for 12 h before surgery and underwent the same anesthetic protocol. The weight of each dog was measured using the same digital scale, and the body condition score (BCS) was assessed and documented by the same veterinary anesthetist using the scoring system from 1–9 of the World Small Animal Veterinary Association (30). Following the allocation to the treatment groups, each dog received the premedication consisting of 20 μg/kg medetomidine (Domitor^®^ 1 mg/mL, Vetoquinol, Switzerland) and 0.3 mg/kg butorphanol (TORBUGESIC^®^, 10 mg/mL, Zoetis, Turkey) intramuscularly administered into the lumbar paravertebral muscles. Five minutes after sedation a venous catheter was placed under sterile conditions into the cephalic vein and the surgical field was clipped, cleaned, and prepared aseptically. After catheter placement, each dog received once a dose of 0.2 mg/kg meloxicam (Metacam, 5 mg/mL, Boehringer Ingelheim, Switzerland) and 0.5 mg/kg metoclopramide (Metamapid, 5 mg/mL, Sifar, Turkey) subcutaneously to control inflammatory pain and minimize the occurrence of perioperative nausea and vomiting. Additionally, each dog received 20 mg/kg amoxicillin (Vilamoks LA, 150 mg/mL, Vilsan, Turkey) subcutaneously to reduce the risk of perioperative infections. For infusion therapy, each dog received Ringer’s lactate (RINGERLACTAT, 250 mL PP-Btl, Bichsel, Switzerland) at a rate of 5 mL/kg/h through the cephalic vein catheter for infusion therapy throughout the whole surgery. Ten minutes after intramuscular injection the EA and sham treatment (Figure 1) was started. The anesthesia of each dog was performed and monitored by the same experienced veterinary anesthetist, who was unaware of the treatment allocation. In both groups general anesthesia was induced with 2 mg/kg ketamine (Ketaset^®^, 100 mg/mL, Zoetis, Turkey) intravenously. All dogs received oxygen flow-by (3 L/min) with a mask during the whole procedure. Any dogs exhibiting signs of nociception or insufficient anesthesia depth received an intravenous bolus of 0.5 mg/kg propofol (Fresenius, 200 mg/20 mL, Switzerland) and 0.5 mg/kg ketamine (Ketaset^®^, 100 mg/mL, Zoetis, Turkey). The intervention was administered if the dog showed at least one of the following signs: a sudden increase of more than 20% from baseline heart rate, a 20% increase in respiratory rate, signs of awakeness, or spontaneous movements. To determine a 20% increase, we used the baseline values collected at timepoint 1 (T1) following induction. Anesthesia monitoring included measurement of arterial saturation of hemoglobin with oxygen (SpO2) using pulsoxymetry, respiratory rates using a capnograph placed in the nasal vestibule (Rad-97^®^ Pulse CO-Oximeter^®^ with NomoLine Capnography, Masimo, United States), heart rate using electrocardiography (ECG) and arterial blood pressure using noninvasive oscillometric measurement (CARESCAPE™ ONE Monitoring System (CS1), GE Healthcare, Germany) and rectal body temperature using a digital thermometer (Covetrus, Switzerland). The mentioned parameters were recorded for each dog every 5 min and additionally at specific time points, namely after induction (=T1), after skin incision (=T2), during traction of the first ovary (=T3), during traction of the second ovary (=T4) and after the first skin suture (=T5).

Ovariohysterectomy was performed 2 min after general anesthesia induction by the same experienced surgeon in each dog, using a midline surgical approach in dorsal recumbency. During the recovery period 5 mg/kg praziquantel (Teniacid, 75 mg/mL, Santavet, Turkey) and 0.2 mg/kg ivermectine (Biomec, 10 mg/mL, Czech Republic) was given subcutaneously to each dog to ensure comprehensive deworming of the dogs.

### 2.4 Electroacupuncture treatment

**Figure 2:**
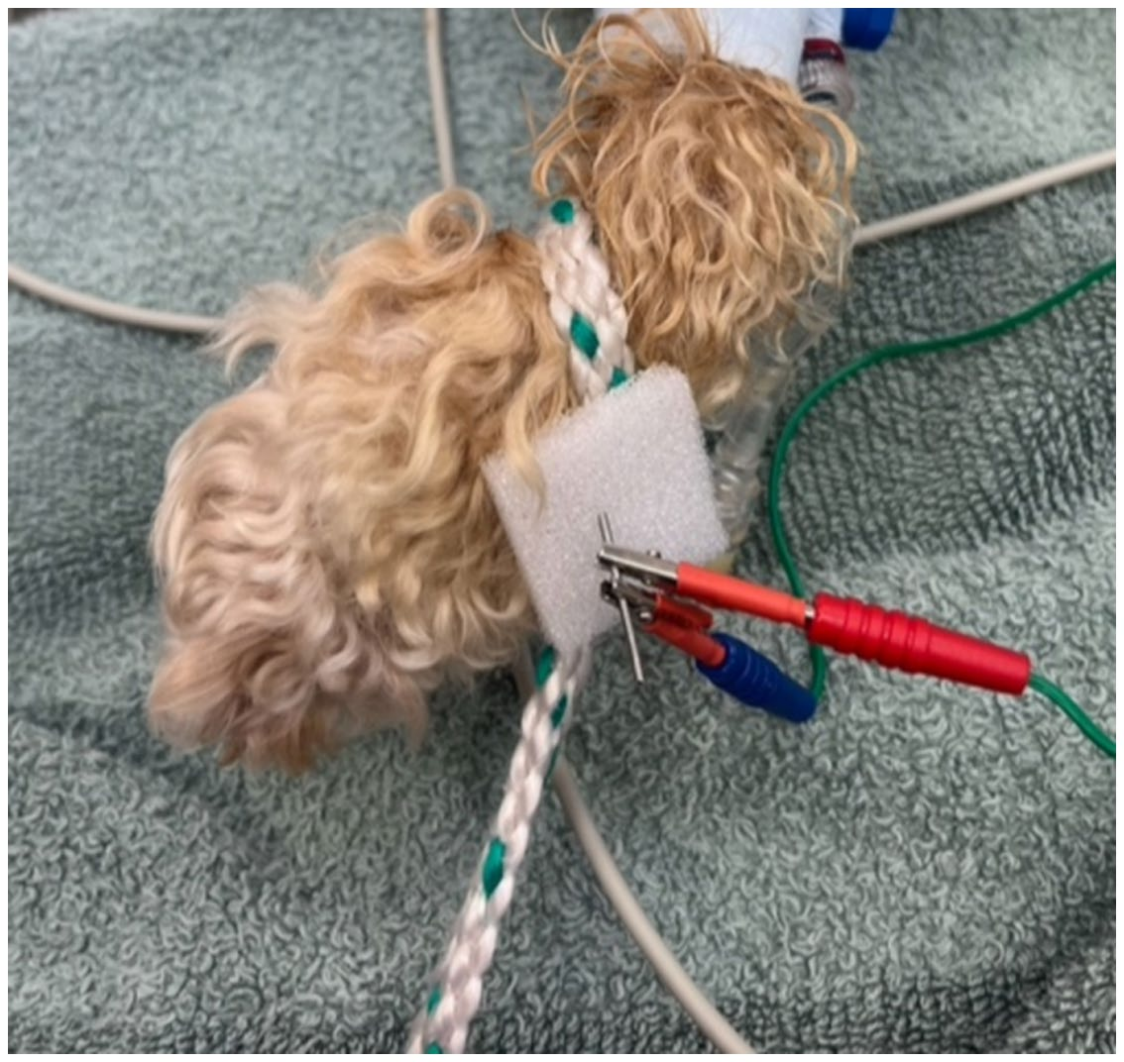
Self-adhesive foam-wound pads (Animal Polsters, Snögg, Switzerland) covering acupoints were used in both groups to ensure blinding of the anesthetist.

Dogs of the EA group received 10 min after sedation the EA treatment by inserting needles (silicone-coated steel needles, 0.18 × 15 mm (< 20 kg), 0.20 × 25 mm (> 20 kg), AcuTop, Germany) to following acupoints bilateral: LI-4 (Large intestine 4), LIV-3 (Liver 3), ST-36 (Stomach 36), SP-6 (Spleen 6), depicted in Figure 1 (31). To ensure blinding of the anesthetist, self-adhesive foam-wound pads (Animal Polsters, Snögg, Switzerland), as depicted in Figure 2, covering acupoints were placed in each dog, so it was not visible whether the electroacupuncture needle was penetrating the skin (EA group) or not (control group).

Ten minutes after sedation, until the end of the surgery procedure, the acupuncture points were electrically stimulated with a JM-3A electroacupuncture stimulator for animals at a frequency of alternating 2 and 100 Hz, titrating the intensity until slight muscle twitches became visible. The frequency was alternated between 2 and 100 Hz every 2 min, to stimulate the release of different transmitters (2 Hz: endorphin, enkephalin, 100 Hz: dynorphin). In the control group the needles were inserted through the foam, but did not penetrate the skin, and were connected to the electrical stimulator as well, like in the EA group, but no current was applied.

### 2.5 Postoperative pain assessment

Postoperative pain was assessed by two experienced and independent veterinary anesthetists, who were blinded to the treatment groups, using the Short Form of the Multimodal Glasgow Composite Pain Scale (mGCPS-SF) presented in the Supplementary material (32). Pain was scored before sedation (T0), and three (T3), six (T6) and 24 h (T24) after anesthesia induction. The mGCPS-SF evaluates six behavioral categories with associated descriptors: vocalization, attention to the wound, mobility, response to touch, demeanor, and posture/activity with a maximum score of 24. According to Reid et al. (32), if dogs pain scores exceed the threshold, which is defined as a score greater or equal to 6/24 the need for rescue analgesia is indicated. To evaluate the level of sedation in the dogs, the validated sedation scale by Grint et al. (33) and Wagner et al. (34) was performed always after the pain score, by the same two blinded anesthetists, before sedation (T0), after sedation (T0 postSed) and three (T3), six (T6) and 24 h (T24) after anesthesia induction.

### 2.6 Statistical analysis

The data were statistically analyzed using the commercial software R and the package for ordinal data in R (35, 36). First, the data were tested for normal distribution, using a Shapiro–Wilk test, as well as graphically. Secondly, linear models for the correlation of the individual variables were performed. After various models were tested for dispersion, a Zero-Inflated Poisson Model was used. The zero-value model made it possible to model data with a significantly increased number of zero values; zero inflation was recognizable from the density function, among other things. The selected variables were total pain scores, treatment (EA, C), time points (0, 3, 6, 24 h), weight, body condition score (BCS), re-injected ketamine and propofol dose, duration of procedure, observer, and sedation score. Interaction terms were tested as well. The significance of the observer variability was tested, using a Friedman test. The significance level was set at *α* = 0.05 for all analyses.

## 3 Results

In total, 71 dogs participated the study (EA, *n* = 34), (C, *n* = 37), of which 56 dogs (EA, *n* = 27), (C, *n* = 29) were included in the study, while 15 dogs (eight in the control group and seven in the EA group) had to be excluded due to previous castration (*n* = 9), late pregnancy (*n* = 2), intraoperative excessive bleeding (*n* = 1), underlying systemic diseases (*n* = 1) and brachycephalic appearance (*n* = 2).

The average weight of the dogs was 16.25 kg (ranging from 5 to 33 kg), and the average body condition score was 4.75 (ranging from 3 to 7), the age was estimated in all dogs above 6 months.

The surgery duration did not differ significantly between the groups, the mean duration was 9.96 min (± 2.35). The mean electroacupuncture treatment duration, depending on the surgery duration, was 11.96 min (± 2.35).

**Figure 3:**
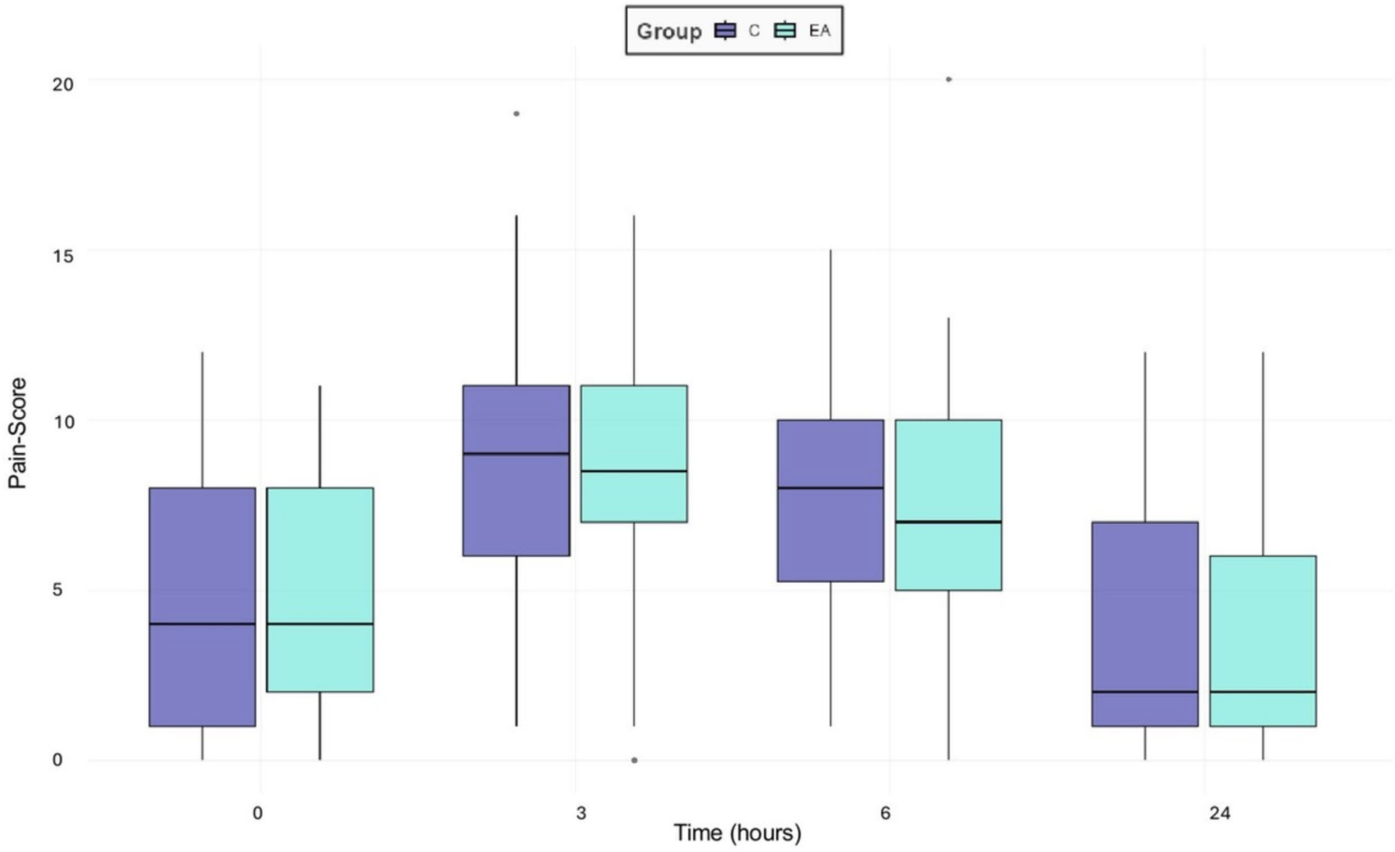
Boxplots presenting the overall median pain scores of 56 female dogs undergoing ovariohysterectomy receiving an additional electroacupuncture treatment (EA) or a sham treatment (C) using the mGCPS-SF (scale 0–20) performed preoperatively (0), three (3), six (6) and 24 h postoperatively. Outliers are marked as individual points in the boxplot.

The Shapiro–Wilk test was highly significant (*p* < 0.01) for both groups and observers, indicating that the values for the pain scores were not normally distributed. There were no significant differences in pain scores among the groups (*p* = 0.36), but among time points, as shown in Figure 3. In both groups, pain scores increased significantly during the early postoperative period (T3 and T6) and decreased after 24 h (*p* = 0.01). The greatest increase of pain scores, by 49%, was observed 3 h after anesthesia induction (*p* = 0.001), whereas after 6 h pain scores were only increased by 39% (*p* = 0.001). Three hours after surgery (T3) 89.4% and 6 h postoperatively (T6) 71.4% of the dogs in both groups exceeded the analgesic intervention threshold of the mCMPS-SF, which is defined as a score greater than or equal to 6/24, indicating the need for rescue analgesia.

**Figure 4:**
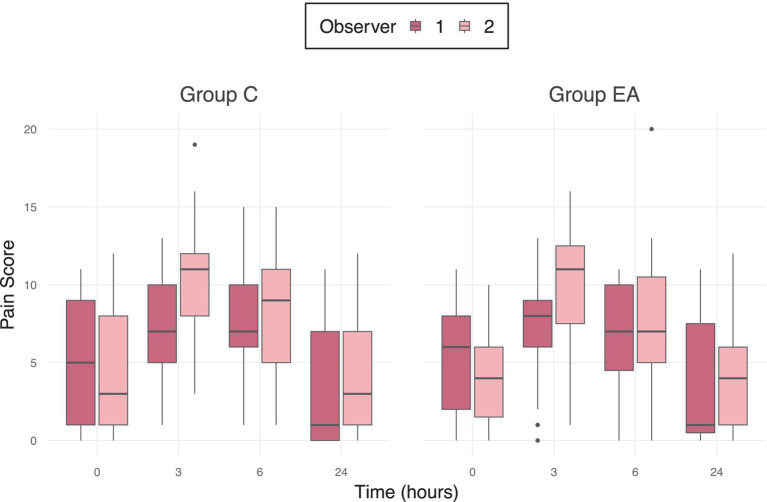
Median pain scores of 56 female dogs undergoing ovariohysterectomy receiving an additional electroacupuncture treatment (EA) or a sham treatment (C) between two different observers using the mGCPS-SF (Modified Glasgow Composite Pain Scale—Short Form; scale 0–20) at preoperative baseline (0 h) and at 3, 6, and 24 h postoperatively. Outliers are marked as individual points in the boxplot.

A significant inter-observer variability in pain scores was observed in both groups (p = 0.001), shown in Figure 4.

There were no significant interactions between pain scores and further variables like weight (*p* = 0.436), body condition score (BCS) (*p* = 0.515), surgery duration (*p* = 0.810) and number of re-injections (p = 0.810) during anesthesia.

## 4 Discussion

According to the results, 10 min of perioperative electroacupuncture did not provide additional complementary postoperative analgesia to conventional analgesic treatments in female dogs undergoing ovariohysterectomy.

In contrast to our results, previous studies have demonstrated a significant analgesic effect of acupuncture treatments in dogs, when compared with the administration of butorphanol alone. Dogs that received an electroacupuncture treatment 40 min prior to the ovariohysterectomy showed lower pain scores, especially within the first 5 h postoperatively, as well as higher plasma *β*-endorphin concentrations 1 h after the surgical procedure (37). Whereas studies that investigated the analgesic effect of electroacupuncture in cats undergoing ovariohysterectomy (38, 39) and dogs referred for mastectomy (40) determined no significant reduction in postoperative pain. Animals in these studies were electrically stimulated for only 20 min or less before surgery, which contrasts with the significantly longer stimulation period in the first mentioned study. In the present study, the anesthesia time was shorter than expected, due to the very experienced surgeon and, thus, the very short operation time. This resulted in shorter surgery durations and, consequently, brief electroacupuncture treatments averaging approximately 10 min. The duration of the applied acupuncture in the present study might have been not sufficient to enable the complete acupuncture related analgesia, as the relevant mechanisms for achieving analgesia require time to take effect, known as induction time. On the peripheral side, activation of the hypothalamus-pituitary–adrenal axis increases corticosterone levels and suppresses proinflammatory cytokines (19–21). Additionally, stimulation of sympathetic nerve fibers induces the release of *β*-endorphin and enkephalin from immune cells in the periphery (16–18) and promotes the release of opioids, norepinephrine, and serotonin in the spinal cord (22, 23), as well as endorphins in the upper brainstem. It is known that it takes a certain amount of time, until a sufficient amount is released to produce an analgesic effect.

Theories based on traditional Chinese medicine, as well as modern western approaches, assume that acupuncture requires 15 to 30 min to develop the full analgesic effect (41, 42). Clinical trials in human medicine support this assumption, as postoperative pain scores after gynecologic surgeries were significantly lower in patients who received an electroacupuncture treatment, when the stimulation was applied for 150 min (43), whereas electroacupuncture treatments with a duration of 20 min or less could not provide any beneficial impact on postoperative pain (44, 45). Based on these studies, we suspect that the acupuncture treatment needs to be at least 20–30 min to develop its full analgesic effect.

A study that investigated whether preoperative or postoperative acupuncture is as effective as meloxicam in relieving pain in dogs undergoing ovariohysterectomy, determined no significant differences for postoperative pain scores and rescue analgesia requirements among the three groups, thus demonstrated the analgesic effectiveness of acupuncture treatments, despite the acupuncture was applied for only 20 min before surgery (46). The results of the mentioned study suggest that, besides the application duration, the timing of acupuncture treatment is a crucial factor influencing the effectiveness and the overall outcome. The concept of preemptive analgesia, which is widely accepted in human and veterinary medicine, aims to prevent central sensitization, induced by painful stimuli, through the implementation of an analgesic intervention prior to the noxious stimulus (47). Preventing the initial sensitization can reduce the response to future nociceptive stimuli, thereby facilitating a less intense perception of typically painful stimuli and decreasing the occurrence of postoperative hyperalgesia and allodynia (48). Clinical trials confirmed that the administration of certain analgesic drugs prior to the painful sensation has a beneficial impact on postoperative pain (49, 50). Furthermore, studies in human medicine also indicate that preemptive applied electroacupuncture results in less postoperative opioid consumption (51) and lower postoperative pain scores (52) compared to electroacupuncture administered after surgery (53). These findings are inconsistent with the results of our study, as the electroacupuncture treatment was also performed in each dog before and during the pain causing procedure.

Another factor that influences the analgesic efficacy and might explain the poor outcome in the present study, is whether the acupuncture treatment is applied to a conscious patient or, as in this case, during general anesthesia. Several imaging studies in human medicine, that investigated brain activity after acupuncture stimulation in awake patients using magnetic resonance imaging (MRI) scans, showed that acupuncture led to both, either significantly decreased or increased functional magnetic resonance imaging (fMRI) signals in the limbic system and subcortical structures, including the nucleus accumbens, amygdala, hippocampus, hypothalamus, and anterior cingulate gyrus, depending on the stimulation intensity (54, 55). This neurophysiological response to acupuncture was significantly impaired, if the treatment was administered during propofol induced anesthesia (56), suggesting that general anesthesia interferes with the acupuncture related modulation of deep brain structures, particularly in regions associated with emotional regulation and pain processing, thus reducing the analgesic effect. Since the dogs in this study were street dogs, characterized by an anxious and nervous nature with a low compliance, we decided that administering the electroacupuncture treatment without any sedation would not be practicable and not ethically justified. Even though some other clinical trials have reported a positive outcome for postoperative pain in dogs following acupuncture treatments that were also performed under deep sedation (37, 46), in this study, the use of sedatives might have diminished the analgesic effectiveness, especially given the brief duration of the treatment. However, it should be considered that the induction time was probably too short to achieve a sufficient release of endogenous neurotransmitters before start of surgery.

In order to obtain relevant results, that are applicable for the private practice in veterinary medicine, a standard anesthetic protocol was used, consisting of ketamine, medetomidine, and butorphanol. Ketamine, a dissociative anesthetic agent commonly used in veterinary medicine, induces a cataleptic state by dissociating the limbic system and thalamus. It is classified as a non-competitive NMDA-receptor antagonist and affects the central sensitization of pain by impeding modulation and transmission of pain perception from the dorsal horn in the spinal cord to upper centers (57, 58). In addition, ketamine has a further agonistic effect on opioid receptors and, when administered intramuscularly in dogs, provides analgesia for approximately 20 to 60 min and reduces postoperative analgesic consumption (59). Some studies have hypothesized that the use of ketamine might interfere with the autonomic nervous system and neurochemical pathways involved in electroacupuncture, potentially diminishing its efficacy (60, 61). However, the specific interaction between ketamine and electroacupuncture is not well-documented or universally agreed upon in the scientific literature. Alpha_2_-adrenoreceptor agonists, like medetomidine, are known to have some antinociceptive properties, that can last up to 115 min in dogs, in addition to their sedative effects (62). Activation of alpha_2_-adrenoreceptors, located in the locus coeruleus in the upper brainstem, causes sedation, whereas the antinociceptive effect is attributed to an intervention in different points of the pain pathway, by inhibiting the nociceptive transmission of primary afferent fibers, affecting the modulation of nociceptive signals in the dorsal horn of the spinal cord, and supraspinally, by influencing modulation systems in the locus coeruleus (12). Butorphanol is a *μ*-opioid receptor antagonist and provides sedation and mild visceral analgesia through an agonistic action on *κ*-opioid receptors, which are primarily located in the spinal cord. In contrast to pure μ-opioid receptor agonists, butorphanol is characterized by a lower analgesic potency and a shorter antinociceptive duration, with up to 60–120 min in dogs compared to methadone (63, 64). Because butorphanol provides partial antagonistic actions at μ-opioid receptors, it could potentially interfere with the μ-opioid mediated effects that are part of acupuncture’s analgesic mechanism. Some studies have shown that opioid antagonists, like naloxone, can modulate or block acupuncture-induced analgesia, by disrupting endogenous opioid pathways, although clear evidence of butorphanol “blocking” acupuncture is limited (65). From these pharmacokinetic data, it can be concluded that, after 2 h, only the non-steroidal anti-inflammatory drug (NSAID) meloxicam was still acting and providing analgesia. With the significant increased pain scores, exceeding the threshold value in over ¾ of the dogs 3 and 6 h postoperatively, it can be deduced that meloxicam alone did not provide sufficient analgesia and rescue analgesia would have been required (32). To simulate realistic conditions occurring in spay projects, we decided against administering rescue analgesia. The evaluation of postoperative pain, using pain scores and providing additional analgesics, is not common practice in such projects and allowed us to assess the duration of the pain. These findings suggest that this widely used anesthetic protocol is not guaranteeing an adequate pain control for the early postoperative period. The peak of pain, following ovariohysterectomy in dogs and cats, is assumed to occur within the first 6 h after surgery, whereas pain scores return back to their baseline values after 20 to 24 h after the surgical procedure (10, 66), emphasizing the need to revise the currently practiced approach of managing postoperative pain after spaying. A systematic review, investigating common analgesia practices in dogs undergoing ovariohysterectomy, reported that the preoperative administration of a single dose opioid is the most preferred method to treat postoperative pain. Although this approach is associated with the highest requirements for rescue analgesia, likely due to the short duration of action of various opioids, better outcomes can be achieved, when analgesic drugs, in particular opioid—NSAID combinations, are administered before and once after surgery. However, opioids are primarily used preoperatively, while for the postoperative period the single treatment with NSAIDs is most popular (67).

Since the majority of dogs in the present study showed increased pain scores during the first 6 h, despite a very experienced surgeon and all receiving meloxicam, a potent NSAID, it suggests that NSAIDs alone may not be sufficient to cover acute postoperative pain completely after an ovariohysterectomy. To ensure a comfortable recovery of our patients and detect these dogs that are suffering from pain, it would be preferable to assess the dog’s pain during the early postoperative period and before discharge from clinics, using a validated pain score. If the scores exceed the defined threshold, the administration of rescue analgesia is indicated. For this purpose, for example, a longer acting opioid, such as buprenorphine, would present a reasonable and suitable choice. In contrast, repeated postoperative NSAID treatments over several days may not be indicated, as pain scores in the present study, as well as in others, return to baseline levels within 24 h. In addition, it is known that the long-term use of NSAIDs carries a high potential to cause severe side effects, such as gastrointestinal lesions, nephrotoxicity, and hypocoagulability (68). These risks can be mitigated by carefully evaluating whether their use is truly indicated and thus justified or if there are alternative methods.

To assess the pain level during the postoperative period, the mGCPS-SF was used in the present study. This behavioral pain assessment tool was developed to evaluate acute postoperative pain in dogs in a clinical setting and involves 6 behavioral categories: vocalization, attention to wound, mobility, response to touch, demeanor, and posture/activity. As the evaluation is based on behavioral traits, several factors may influence the outcome (32). Besides demographic factors, the individual personality and the presence of fear or anxiety have a major impact on how pain is expressed and perceived by the observer (69). A clinical trial was able to show that pain scores in shelter cats following orchiectomy were postoperatively significantly higher in cats that were, based on a demeanor score, categorized as friendly, whereas anxious and aggressive cats did not show increased postoperative pain scores compared to their baseline values, demonstrating that demeanor influences the pain assessment (70). The unknown interaction with and handling by humans evoked an anxious and insecure behavior in most of the dogs, which likely resulted in an overestimation of pain scores, as indicated by elevated scores observed in approximately 45.6% of the dogs even at the baseline assessment before surgery. The findings align with previous studies, that investigated pain in canine radiation patients, and reported a similar influence of anxiety on pain score outcomes (71, 72) and demonstrated that dogs with extrovert personalities expressed pain more clearly (73). Since we observed a significant decrease in pain scores in both groups 24 h after the surgery, we assume that the dogs adapted over time and became accustomed to the new environment and handling.

The mGCPS-SF was developed and validated in several languages to evaluate acute pain in client owned, domesticated dogs, providing a reliable and consistent pain assessment tool for trained clinicians (32, 74, 75). Since feral dogs are not accustomed to any handling, they tend to show overstated responses, for example, tense abdomen or snapping, they refuse to move, as they are not familiar with walking on leashes and appear anxious to the observer. Considering that these behaviors may result in higher scores and might cause that anxiety is unintentionally interpreted as pain, the latest mGCPS-SF is most likely strongly biased to assess acute pain in undomesticated feral dogs and should be specially validated and possibly adapted for this purpose. Another factor that influences pain perception and its expression is gender. In humans, women tend to have lower pain thresholds, compared to men, and experience higher pain perception (76). This can be attributed to the effects of steroid hormones, such as estrogen, on the nervous system. These hormones modulate neurotransmitters in the brain, spinal cord, and peripheral nerves and alter the excitability of specific brain regions, which can impact the pain perception (77). Clinical studies that have examined pain perception in male and female dogs also support this observation, as intact female dogs have shown higher pain scores compared to males (78). Conducting our study exclusively with intact female dogs may have influenced the overall outcome of the pain scores; however, it did not affect the comparability between the two groups.

The difficulty of accurately assessing pain in feral dogs, using currently available behavioral scoring systems, is potentially the main limitation in the present study. The capture of the dogs and their hospitalization in an unfamiliar environment subjected them to straining conditions, that caused significant stress for each dog and resulted in a fearful demeanor. Since fear in dogs leads to behavioral expressions similar to those of pain (72), it can significantly impact the pain assessment and, as demonstrated in the present study, result in notably elevated scores, both before and after surgery. The consequence is that minor changes in pain scores during the early postoperative period, considered the most critical phase for evaluating postoperative pain, may not be detected, whereas dogs tend to adapt after a certain acclimatization time and subsequently express their emotional state more clearly.

The significant interobserver variability also limits the interpretability of the results and suggests that even validated pain assessment tools are susceptible to fluctuations, influenced by factors such as the observer’s gender, previous experience, and level of training (79). Despite the significant difference between scorers, the trend is consistent, and both scorers arrived at a score above the threshold at 3 and 6 h postoperatively. Interestingly, the second observer did not score higher only preoperatively, but at all other time points, which could indicate a possible influence of the knowledge, that the dogs had not yet undergone surgery, as the pain observers were not blinded to the evaluation timepoints. Figure 3 also shows, that both observers recorded greater variability in pain scores during the baseline assessment and 24 h after anesthesia — when the dogs’ behavior was not influenced by any anesthetics — than during the early postoperative period, when their natural behavior may still have been dampened by the anesthetic drugs. This also emphasizes the role of behavior in pain assessment, particularly in anxious dogs, and indicates that pain evaluation, using currently available behavioral assessment tools, is challenging and may be strongly biased. It is necessary to consider, however, that the applied pain assessment tool has not been validated for feral dogs and may explain the results in the present study. Since the study was conducted as part of a catch-neuter-release program, there were considerable demographic variations among the dogs, such as age, weight, and particularly the BCS. These differences in fat and muscle distribution may have influenced the pharmacodynamics and duration of action of administered anesthetics, though a statistically significant correlation could not be determined.

Furthermore, timing and duration of the electroacupuncture treatment were not preferable to fully evolve the beneficial and pain-relieving effect of acupuncture. Given the short surgery duration, the dogs were stimulated for only 10 min on average, while previous data indicate that acupuncture requires an induction time of 15 to 30 min to reveal the complete analgesic effect. Along with the unfavorable timing of the treatment, namely when the dogs were already sedated and unconscious, the analgesic effect might have been diminished and could explain the poor results. To further investigate the analgesic properties of acupuncture and its effectiveness for managing postoperative pain in dogs, additional studies are needed. These studies should take into account the individual temperament of the dog, when assessing pain or consider using modified behavioral assessment tools. Moreover, ensuring a sufficiently long duration of acupuncture treatment, preferably in unsedated dogs, would be advantageous.

## 5 Conclusion

In the present study, a supplementary electroacupuncture treatment, perioperatively administered for 10 min, did not improve postoperative pain in dogs undergoing ovariohysterectomy. However, it was shown that over ¾ of the dogs exceeded the pain score threshold in the early postoperative phase and the widely used analgesic protocol was, therefore, insufficient in most dogs. The impact of anxious behavior on pain evaluation in dogs, using the mGCPS-SF, can significantly influence the outcome. To ensure an adequate postoperative pain management and detect dogs that are suffering from pain during the postoperative period, pain should be evaluated, and rescue analgesia administered if required, as a standard anesthesia protocol consisting of butorphanol, ketamine, and medetomidine may not provide a long lasting and sufficient pain relief.

## Data Availability

The raw data supporting the conclusions of this article will be made available by the authors, without undue reservation.
